# The genetic architecture and spatiotemporal dynamics of adaptation across human‐modified landscapes

**DOI:** 10.1111/nph.70520

**Published:** 2025-09-08

**Authors:** Julia M. Kreiner

**Affiliations:** ^1^ Department of Ecology & Evolution University of Chicago Chicago IL 60637 USA

**Keywords:** fluctuating selection, genetic architecture, genomic timeseries, migration, parallel evolution, polygenic, rapid adaptation

## Abstract

Understanding the rate and nature of adaptation is crucial for managing biodiversity across our changing landscapes. This perspective synthesizes insights from resistance evolution – a case of rapid, repeated adaptation to extreme human‐mediated selection – to reveal how adaptive genetic architectures determine and feedback with evolutionary dynamics. Recent population genomic and quantitative genetic approaches have demonstrated that the extent of genetic parallelism and reliance on *de novo* vs standing genetic variation can vary with the complexity of genetic architectures. However, we are only starting to understand how spatial and temporal heterogeneity influence the importance of alternative genetic architectures within and among populations, and the pace of adaptation across scales. I outline how the integration of landscape‐scale population genomics with high‐resolution genomic time series has the potential to transform our understanding of these phenomena. With careful consideration of their limitations, spatiotemporal approaches should prove powerful for reconstructing and predicting the adaptive dynamics of populations across increasingly variable geographic landscapes — from pesticide resistance to climate adaptation.

**Table:** 

	Contents	
	Summary	744
I.	Introduction	744
II.	The genetic architecture of resistance and its implications for predicting adaptation	745
III.	The promise of spatiotemporal genomics for direct observation of evolutionary dynamics	746
IV.	Conclusions	748
	Acknowledgements	749
	References	749

## I. Introduction

The survival of endangered species and our ability to combat the spread of pests hinge on a fundamental question in evolutionary biology: what limits the speed of adaptation in a changing world? A key rate‐limiting contributor is the underlying genetic architecture; the number of required genetic changes and their individual effects across environments dictate whether populations can maintain sufficient standing variation and incorporate new mutations fast enough to avoid extinction (Barrett & Schluter, 2008). In large populations where both mutational input and standing variation are high, adaptation should proceed rapidly and repeatedly under strong selection (Gillespie, 1983; Karasov *et al*., 2010; Kreiner *et al*., 2022b). While this demographic context contrasts sharply with that of endangered species, it often characterizes pest species, allowing for the use of population genomic approaches that rely on deterministic evolutionary dynamics. Yet, even with these deterministic dynamics, the genetic mechanisms underlying adaptation vary considerably between populations, suggesting that spatiotemporal variation in selection pressures across heterogeneous landscapes may shape both the genetic architecture of adaptation and the pace at which it unfolds.

In this perspective, I synthesize our understanding of how these adaptive dynamics play out over contemporary scales, focusing on tractable systems where human‐mediated evolution occurs rapidly and genetic architectures can be precisely dissected – adaptation to pesticides. Pesticides represent one of humanity's most potent selective forces, with 32% of global land used for agriculture, and pesticide use intensifying in these environments over recent decades (Fuglie *et al*., 2024). In response, resistance has evolved repeatedly in persisting populations (Heap, 2022). Natural populations not experiencing pesticide use exist in these landscapes with varying connectivity to treated populations, creating migration‐selection balance dynamics that influence resistance evolution (Otto & Bourguet, 1999). Recent work has begun to reveal how heterogeneous selection regimes – such as spatial variation in use, temporal rotations, and pesticide mixtures – influence whether single variants with large effects or many variants across the genome, each with small effects (polygenic architectures), underlie adaptation. To capture these dynamics as they occur, breakthrough methods for curating high‐quality genomic time series from museum and herbarium specimens (Gutaker & Burbano, 2017; Kim *et al*., 2023) now enable investigation of how selective strength, dominance relationships, and gene flow govern the pace of evolution. These approaches offer new insights into how genetic architectures and the speed of adaptation interact across human‐mediated landscapes.

## II. The genetic architecture of resistance and its implications for predicting adaptation

The evolutionary genetics of pesticide resistance is best understood through top‐down approaches that map resistance phenotypes to single nonsynonymous changes in genes encoding known, targeted proteins. Such studies have resolved extreme adaptive parallelism, from identical SNPs to convergent substitutions within and among amino acid positions in the target gene (e.g. Cao *et al*., 2025), with these patterns further replicated across species (Heap, 2022). Because these large‐effect mutations, once selected, leave behind distinctive genomic signatures depending on their age and source (Lee & Coop, 2017), bottom‐up, genomics‐first approaches are increasingly resolving the relative importance of mechanisms facilitating their spread – gene flow, *de novo* mutation, and selection on standing variation (Kreiner *et al*., 2019; North *et al*., 2024). Evidence is mounting for the importance of repeated, *de novo* origins of resistance within and across species (Van Etten *et al*., 2020; *Cao et al*., 2025). However, standing variation for large‐effect alleles has been implicated in resistance evolution (Kersten *et al*., 2023) and historical data have allowed for direct observation of these variants before the onset of selection (Baucom & Mauricio, 2010; Délye *et al*., 2013). Since mutational input depends on the census population size (*N*) while standing genetic variation depends on the effective population size (*N*_*e*_), evaluating these mechanisms across species with variable reproductive modes and dispersal strategies that decouple *N* from *N*_*e*_ will be critical for understanding constraints on the pace of adaptation (Kreiner *et al*., 2018). This applies broadly across pesticide resistance systems, as insecticides, herbicides, and fungicides all interact with target species biology in informative ways (Hawkins *et al*., 2018), despite my focus on plants here. For example, in the plant *Amaranthus tuberculatus*, a large contemporary census population size across the landscape is associated with a remarkable number of recent origins of target‐site resistance (Kreiner *et al*., 2022b), suggesting that even in high *N*_*e*_ outcrossing species, mutational input may be a primary determinant of the availability of large‐effect variants.

Population genomic and quantitative genetic approaches have revealed considerable complexity in the genetic architecture underlying rapid responses to extreme selection from pesticides. Resistance has evolved through compensatory SNPs within target genes (Cohan *et al*., 1994; Yu *et al*., 2015), TE insertions (Montgomery *et al*., 2024; Raingeval *et al*., 2024), copy number polymorphisms driven by tandem amplification (Jugulam *et al*., 2014), and the excision of amplified copies into extrachromosomal circular DNA (Molin *et al*., 2020). Even for these seemingly complex genetic mechanisms, rapid evolution can be driven by repeated mutational origins (Kreiner *et al*., 2019). In morning glory, bottom‐up sweep scan approaches across populations with no target‐site mechanism resolved an oligogenic architecture of resistance with both parallel and nonparallel genetic mechanisms across populations (Van Etten *et al*., 2020). Such approaches are powerful for resolving loci with particularly strong effects on focal traits, but may miss the collective contribution of other loci across the genome that do not produce detectable selective sweeps. Multiple lines of evidence support this broader genomic contribution: experimental evolution of sensitive populations under sublethal herbicide doses rapidly produces continuous trait distributions (Neve & Powles, 2005); field surveys document extensive quantitative, heritable variation in herbicide susceptibility within and among populations (Jasieniuk *et al*., 1996); and genetic crosses show intermediate F1 phenotypes with continuous segregation patterns (Petit *et al*., 2010; Huffman *et al*., 2015). In blackgrass, while large‐effect target‐site mutations are common, multiple quantitative trait locus (QTL) mapping populations revealed co‐occurring polygenic variation at 15 distinct loci that differed among populations (Comont *et al*., 2020; Cai *et al*., 2023). While QTL mapping can separate true pleiotropy from linkage‐based trait correlations, it captures only a subset of the genetic variation underlying adaptation.

To comprehensively map the complex genetic architecture of resistance across populations, genome‐wide association (GWA) studies offer a powerful approach. Sample size limitations typically constrain GWA studies' ability to detect low‐frequency and small‐effect variants; however, strong directional selection imposed by pesticides is, in theory, expected to elevate such alleles to detectable frequencies (Patel *et al*., 2024). Building on this framework and the observation that large‐effect target‐site alleles explained only a minority of glyphosate resistance variation in *A. tuberculatus*, we conducted a multivariate GWAS controlling for target‐site status. This identified several hundred additional resistance‐correlated alleles that, while heterogeneous across populations, collectively explained nearly as much resistance variation as target‐site alleles (Kreiner *et al*., 2021). Results from resistance studies so far therefore support the prediction that polygenic architectures involved in local adaptation should be less prone to genetic parallelism than monogenic architectures (Barghi *et al*., 2020). Understanding the importance and predictability of adaptive mechanisms across species will require sustained efforts to detect genetic architectures spanning the continuum of allelic effect size and frequency.

Given the documented variation in both the presence and co‐occurrence of very large‐effect alleles alongside polygenic architectures across adaptive contexts (Sinclair‐Waters *et al*., 2020; Lopez‐Arboleda *et al*., 2021; Neto & Hancock, 2023), several fundamental questions regarding their dynamics remain unclear: Do large‐effect variants always evolve first after distant shifts in the fitness optimum (i.e. such as herbicide applications; Kopp & Hermisson, 2007)? Do small‐effect variants tend to subsequently follow to avoid overshooting the optimum (Orr, 1998; as demonstrated for antibiotic resistance, MacLean *et al*., 2010)? Do shifts in their relative distribution across the landscape reflect pre‐adaptation (e.g. Kreiner *et al*., 2022a), where the architecture of available standing genetic variation depends on historical selection by different environmental stresses? Are the trajectories of polygenic architectures more constrained by fluctuating selection regimes than those of oligogenic or monogenic architectures (Yeaman & Whitlock, 2011)? Resolving these dynamics can be achieved by capturing them as they unfold with spatiotemporal genomic data.

## III. The promise of spatiotemporal genomics for direct observation of evolutionary dynamics

The rise of contemporary, historical, and ancient time series DNA sequencing has transformed our ability to track adaptation in real time. While evolve‐and‐resequence experiments have long been implemented in the lab to study adaptive dynamics (e.g. Barrick & Lenski, 2009), observation of these dynamics in natural populations across realistic landscapes is key to understanding relevant evolutionary responses to environmental change. However, certain limitations persist, and methodological considerations in working with these data merit recognition.

**Fig. 1:**
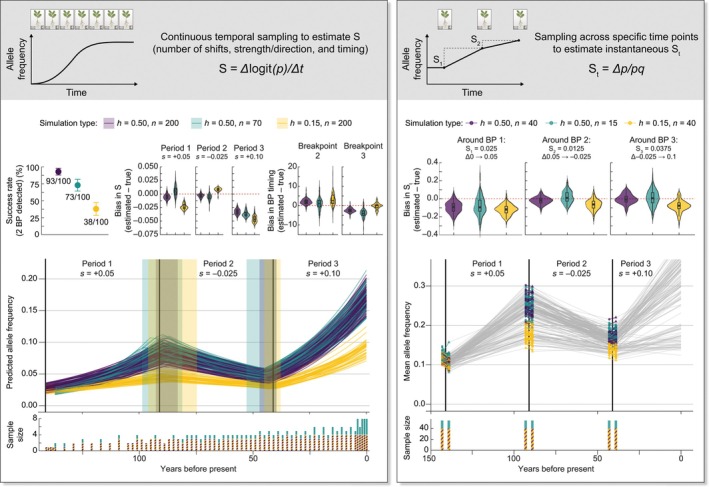
Temporal genomic approaches for measuring selection coefficients from allele frequency change vary in power and accuracy depending on sampling design, sample size, and dominance. *SLiM design*: Forward genetic simulations modeled a population in 2D space evolving under temporally fluctuating selection, similar to contemporary herbicide management regime shifts. Simulations included three selective periods: Period 1 (mean *s* = +0.05), Period 2 (mean *s* = −0.025), and Period 3 (mean *s* = +0.10; all following an exponential distribution of fitness effects), with shifts in selection (breakpoints, BPs) occurring at generations 50 and 100 after a 2000 generation (1 generation yr^–1^) neutral burn‐in (BPs in years before present/last sampling = 2141). A 100 kb region was simulated, with a mutation rate of μ = 5 × 10^−6^, a recombination rate *r* = 2.3 × 10^−5^, and an *n* = 5000. One hundred replicate simulations were performed for each scenario. *Left panel*: Continuous temporal sampling allows for logistic regression inference of predicted allele frequency trajectories over time, where sampling intensifies over recent timescales in a fashion similar to museum and herbarium collections. Three simulation scenarios were tested that vary dominance and sample size: (1) *h* = 0.5, *n* = 200; (2) *h* = 0.5, *n* = 70; and (3) *h* = 0.15, *n* = 200. Each trajectory represents the logistic predicted value of all known selected loci (*c*. ~300 per simulation) when analyzed jointly. Vertical black lines mark true BPs between selection regimes. Colored shaded regions show the range (min–max) of estimated BPs across successful simulations, focusing on shifts between nonneutral regimes during the sampled time period (where BP = 2). The upper plots show method performance for detecting selection using breakpoint regression analysis: success rates for detecting the correct number of BPs (left, points with SE bars), bias in estimated selection coefficients for each period (when BPs = 2, center, where violin plots show the estimated distribution shape and embedded box plots show interquartile and median summary statistics), and bias in estimated BP timing (when BP = 2, right). *Right panel*: Discrete sampling before and after a known selective shift (e.g. herbicide introduction, extended drought) illustrates an approach for measuring instantaneous selection. Three scenarios were tested, again spanning dominance and sample size: (1) *h* = 0.5, *n* = 40 × 6 timesteps; (2) *h* = 0.5, *n* = 15 × 6 timesteps; (3) *h* = 0.15, *n* = 40 × 6 timesteps. In the lower middle plot, each point represents the mean allele frequency estimate (*p*) of all known selected loci (*c*. ~300) before and after a known shift in selection regime (vertical black lines). In the upper plots, violin distributions show the bias and accuracy of instantaneous selection coefficient estimation across simulation replicates for three different selection regimes. Code and further details are available at https://github.com/jkreinz/slim‐breakpoint‐analysis.

Temporal genomic data enable precise estimation of the strength and direction of selection (Kelly, 2022; He *et al*., 2023; Akbari *et al*., 2024) and therefore the relevant fitness consequences of genetic variation, though accuracy depends critically on analytical approach and sampling design. Since allele frequency change depends on the product *p*(1−*p*) – maximized at intermediate frequencies – the logit transformation of allele frequency change (*S = ∆*logit(*p*)*/∆t*) provides an intuitive estimate of *s* when the efficacy of selection (*N*_*e*_ *s*) is strong (Crow & Kimura, 2009; Taus *et al*., 2017). Continuous temporal sampling, such as that provided by museum and herbarium collections, with logistic inference has particular advantages: Logistic models can quantify not only selection strength and direction but also the number and timing of selective shifts through breakpoint regression (Fig. 1) and estimate environment‐specific selection coefficients through modeling interaction effects. This approach is well suited for collective analysis of candidate loci (identified through GWAS, or gene–environment associations which can help to detect the selective source; Wade & Kalisz, 1990) as multiple independent observations across loci and time points enable precise averaged parameter estimation (depending on sample size and dominance; Fig. 1; Box 1). By contrast, discrete sampling around known selective shifts provides much less statistical power for detecting (the instantaneous) strength of selection (S_t_ = ∆*p*/*p*(1 − *p*)) (Fig. 1), although replicated designs can improve such approaches (e.g. for resolving parallel responses to changing climate, Wu *et al*., 2025). Even with continuous temporal sampling and logistic inference, shifts in population structure through time or recurrent migration can bias selection estimates and should be controlled for using kinship matrices as a random effect (Akbari *et al*., 2024). Furthermore, when evolution is governed by stochastic dynamics, such as in endangered, small *N*_*e*_ species, methods that explicitly account for genetic drift should be considered (Bollback *et al*., 2008; Steinrücken *et al*., 2014). These methodological considerations highlight the importance of matching analytical approaches to sampling schemes, experimental design, and the species underlying evolutionary history.

Empirical studies of selective responses in natural populations reveal the complexity of these dynamics across different contexts. Logistic regression analyses of herbarium genomic time series demonstrated that herbicide resistance alleles confer strong but variable fitness advantages (*s* = 0.05–0.35) in Midwestern *A. tuberculatus* populations over the last 60 yr, depending on the specific resistance mutation (Kreiner *et al*., 2022a). These estimates highlight extreme responses to a novel selective pressure despite fluctuating herbicide use through space and time. Importantly, estimates of selection made directly from allele frequency trajectories must also consider that dominance (*h*) mediates this response (Fig. 1; Box 1), with recent approaches focusing on joint estimation of *s* and *h* from genomic time series (Foll *et al*., 2015; Fine & Steinrücken, 2024). The importance of dominance is exemplified by spatiotemporal studies of insecticide resistance in *Drosophila*, which provide strong empirical evidence that pleiotropic trade‐offs across environments with fluctuating pesticide use can result in dominance reversals (recessive costs, dominant benefits) sufficient to fundamentally alter allele frequency trajectories (Karageorgi *et al*., 2024). Similar dominance patterns are reported for several, but not all, herbicide resistance alleles in *Arabidopsis* (Roux *et al*., 2004, 2005), suggesting that fluctuating chemical applications may contribute to the broadscale maintenance of adaptive variation across changing landscapes and could even lead to the evolution of dominance itself, as predicted by theory (Gillespie & Langley, 1974; Otto & Bourguet, 1999). These context‐dependent dynamics, when combined with allelic‐, background‐, and habitat‐dependent fitness costs (Vila‐Aiub *et al*., 2009), suggest that increased spatiotemporal resolution of sampling and parameterization of these dynamics can reveal extreme granularity in selective responses, as documented by Bitter *et al*. (2024).

Box 1 **From allele frequencies to selection coefficients: Why dominance matters**

Basic model: The traditional parameterization of fitness is *w*_aa_ = 1, *w*_Aa_ = 1 + *hs*, *w*_AA_ = 1 + *s*, where *s* is the selection coefficient and *h* is the dominance coefficient. Under deterministic conditions, allele frequency change, $\Delta p=\frac{\mathrm{sp}\left(1-p\right)\left[p+h\left(1-2p\right)\right]}{\underline{w}}$. This expression can be simplified to Δ*p* = *Sp*(1−*p*) by defining *S* = *s*[*p* + *h*(1–2*p*)]/*w̄*, where *S* (here, the effective selection coefficient) captures frequency‐dependent dominance effects, allowing standard haploid selection theory to be applied to diploid systems.

The logit connection: With the haploid form Δ*p* = *Sp*(1−*p*), the logit transformation logit(*p*) = ln[*p*/(1−*p*)] removes the basic frequency dependence, yielding Δlogit(*p*) ≈ SΔT. When dominance is additive (*h* = 0.5), this provides a linear relationship with time, allowing direct estimation of *s*/2 from the slope of allele frequency change through time.

When *h* ≠ 0.5, and *p* ≠ 0.5, the relationship between *S* and *s* breaks down and depends on both dominance and allele frequency:

**Table:** 

Allele frequency	Recessive (*h* = 0)	Additive (*h* = 0.5)	Dominant (*h* = 1)
Rare (*p* ≈ 0)	*S* ≈ *sh* = 0	*S* ≈ *s*/2	*S* ≈ *sh* = *s*
Intermediate (*p* ≈ 0.5)	*S* ≈ *s*/2	*S* ≈ *s*/2	*S* ≈ *s*/2
Near Fixation (*p* ≈ 1)	*S* ≈ *s*(1−*h*) = *s*	*S* ≈ *s*/2	*S* ≈ *s*(1−*h*) = 0

This means recessive beneficial mutations appear nearly neutral when rare but show strong selection near fixation, while dominant beneficial mutations show strong selection when rare but appear nearly neutral when nearly fixed – highlighting the importance of considering dominance when interpreting temporal genomic data.

Understanding how and why genetic architectures themselves evolve is crucial for interpreting these spatiotemporal dynamics. In particular, gene flow and heterogeneous selective regimes can constrain which variants are able to persist under local selection. Herbarium genomics has provided evidence that large‐effect, putative structural variants are important drivers of parallel local adaptation to climate in ragweed (Battlay *et al*., 2023) – consistent with theory predicting that local adaptation should be concentrated at relatively few loci when populations are faced with swamping gene flow from maladapted populations (Yeaman & Otto, 2011). By contrast, increasing selective complexity from single to mixed herbicides has been shown to drive the evolution of generalist, polygenic resistance without detectable fitness costs (Comont *et al*., 2020, 2022). To formally model the dynamics of these adaptive architectures across scales, extensions of the Wright–Fisher diffusion process can estimate how adaptive alleles spread through space and time (Muktupavela *et al*., 2022). These models can therefore identify barriers to allelic spread across landscapes, and when combined with explicit estimation of dispersal and migration rates (Osmond & Coop, 2024; Shastry *et al*., 2025) could help to uncover how gene flow and environmental heterogeneity contribute to the evolution of adaptive architectures.

Leveraging our growing understanding of adaptive architectures, recent work has sought to reconstruct the evolution of complex traits using temporal genomic data in the context of domestication and climate change (Swarts *et al*., 2017; Lang *et al.*, 2024). While genomic trait reconstruction may be accurate for simple architectures like target‐site resistance – where heritability is high and gene‐by‐environment interactions are low – extending genomic prediction through time to complex traits using polygenic scores (PGS = ∑βᵢ*p*ᵢ, where β is estimated allelic effect size and *p* is allele frequency) assumes temporal stability in genotype–phenotype relationships. This assumption may be violated by natural selection driving allelic turnover (Carlson *et al*., 2022), changing gene‐by‐environment relationships (Mostafavi *et al*., 2020), and evolving linkage disequilibrium patterns (Carlson *et al*., 2013). Even when these traits are measurable, phenotypic plasticity can confound validation, as environmental changes can mimic or mask genetic contributions to trait evolution. While ‘virtual common garden’ models attempt to partition plastic vs adaptive contributions to phenotypic time series (Wu & Colautti, 2022), fundamental challenges to the temporal stability of genotype–phenotype relationships persist.

Beyond reconstructing past evolutionary dynamics, genomic time series data offer considerable opportunities to advance genomic forecasting. A widely used approach for predicting population vulnerability to environmental change is to estimate genomic offset – the degree to which current gene–environment associations will be mismatched under future environmental conditions (Fitzpatrick & Keller, 2015). These landscape‐level predictions rely on the critical assumption that associations between environmental variables and genomic data from a single time point reflect a state of local adaptation, and that space‐for‐time substitutions hold (Lind & Lotterhos, 2024). Integrating temporal data into random forest models with explicit spatiotemporal covariates (e.g. Hengl *et al*., 2018) could enable one to dynamically capture populations moving toward their local optima and better incorporate scale‐dependent effects. This temporal framework could shift genomic forecasting from a static vulnerability assessment to dynamic modeling of adaptive responses across changing landscapes.

## IV. Conclusions

The study of rapid and repeated evolution provides a powerful framework for resolving the determinants of the speed and genetic architecture of adaptation. The extreme selection imposed by pesticides can drive predictable evolutionary responses, yet the diversity of genetic solutions across landscapes highlights how spatiotemporal heterogeneity collectively shapes these adaptive outcomes. Advancements in population genomic inference methods and the production of genomic time series from historical landscape‐scale data now enable us to disentangle the interplay between selection and gene flow in shaping the genetic architecture of adaptation across interconnected environments. While the focus here has been on pest populations operating under strong, deterministic selection, insights from these systems can also inform conservation strategies for endangered species where adaptive potential is constrained by small population sizes and stochastic evolutionary dynamics. In particular, the promise of spatiotemporal genomic approaches for assessing the pace of adaptive responses, the role of fluctuating selection, and the importance of migration and dominance in determining evolutionary dynamics across changing landscapes – with the methodological considerations outlined earlier – remain applicable across both contexts. These approaches create opportunities to pursue contrasting management goals: enhancing evolutionary potential to conserve biodiversity while suppressing rapid evolution through pest control. As environmental change accelerates, our ability to predict which populations will adapt, how quickly, and through what genetic mechanisms will determine our success in achieving these objectives.

## Competing interests

None declared.

## Author contributions

JMK was the sole author of this work.

## Disclaimer

The New Phytologist Foundation remains neutral with regard to jurisdictional claims in maps and in any institutional affiliations.
